# Complications after complex device implantation: how important is implanter seniority?

**DOI:** 10.1136/openhrt-2025-003428

**Published:** 2025-09-03

**Authors:** Paul A Scott, Antonio Cannata, Daniel I Bromage, Ian J Wright, Anish Bhuva, Matthew J Lovell, Chris Plummer, Mark de Belder, Mark Dayer, Francis Murgatroyd

**Affiliations:** 1 King’s British Heart Foundation Centre of Research Excellence, School of Cardiovascular and Metabolic Medicine & Sciences, King’s College London, London, UK; 2 National Institute for Cardiovascular Outcomes Research (NICOR), NHS Arden & GEM Commissioning Support Unit, Leicester, UK; 3 Department of Cardiology, Hammersmith Hospital, Imperial College Healthcare NHS Trust, London, UK; 4 Department of Cardiology, Barts Health NHS Trust, London, UK; 5 Institute of Cardiovascular Sciences, University College London, London, UK; 6 Department of Cardiology, Royal Devon and Exeter Hospital, Exeter, UK; 7 Department of Cardiology, Newcastle Upon Tyne Hospitals NHS Foundation Trust, Newcastle upon Tyne, UK; 8 Cardiovascular Research Institute, Mater Private Network, Dublin, Ireland; 9 Faculty of Health, University of Plymouth, Plymouth, UK

**Keywords:** CARDIAC RESYNCHRONISATION THERAPY, Defibrillators, Implantable, Epidemiology

## Abstract

**Background:**

The complication risk of procedures may be influenced by operator and institutional characteristics. Our aim was to assess whether supervising consultant seniority and operative volume, and hospital volume were associated with the risk of reintervention following complex device implantation.

**Methods:**

A nationwide population-based study was performed using the National Institute for Cardiovascular Outcomes Research registry including all patients receiving their first transvenous implantable cardioverter defibrillator or cardiac resynchronisation therapy (CRT) implant in England over 5 years (April 2014–March 2019). The primary endpoint was 1-year reintervention. We evaluated the association between reintervention and supervising consultant annualised complex device volume, supervising consultant seniority and hospital annualised complex device volume, using multilevel logistic regression.

**Results:**

47 630 implants were included. The 1-year reintervention rate was 6.1% (N=2916). There was no difference in reintervention risk with increasing supervising consultant volume (OR 0.89 Q4 vs Q1; 95% CI 0.76 to 1.05, p=0.17). When CRT-pacemakers/defibrillators implants were analysed separately (N=26 108), there was an association between operator volume and 1-year reintervention, but this was of borderline statistical significance and only evident in the highest compared with the lowest volume quartile of operators (adjusted OR 0.79 Q4 vs Q1; 95% CI 0.63 to 0.98, p=0.03). There was a non-linear relationship between reintervention risk and supervising consultant seniority, with the operators in the middle two quartiles of seniority having a lower risk (OR 0.87 Q2 vs Q1, p=0.02; OR 0.81 Q3 vs.Q1; p=0.003) while the most and least senior operators had a similar reintervention risk (OR 0.93 Q4 vs Q1, p=0.31). Hospital volume was not associated with 1-year reintervention.

**Conclusions:**

There is a U-shaped curve between operator seniority and reintervention risk for complex devices. Although there are several potential explanations, these data suggest that while newly qualified consultants may benefit from mentoring, all operators should continuously evaluate their outcomes and share them within their centre and more widely through the national audit.

**WHAT IS ALREADY KNOWN ON THIS TOPIC**

- Operator and hospital characteristics, including procedure volume, are associated with outcomes following complex device implantation. However, less is known about the relationship between operator seniority and outcomes with complex devices.

**WHAT THIS STUDY ADDS**

- In this observational study of 47 630 complex device implants over a 5-year study period in England, there is a U-shaped curve between operator seniority and reintervention risk for complex devices, but neither supervising consultant nor centre operative volume are associated with reintervention risk.

**HOW THIS STUDY MIGHT AFFECT RESEARCH, PRACTICE OR POLICY**

- These data suggest that while newly qualified consultants may benefit from mentoring, all operators should continuously evaluate their outcomes and share them within their centre and more widely through national audit.

## Introduction

 Complex cardiac implantable electronic devices (CIED) (implantable cardioverter defibrillator (ICD) and cardiac resynchronisation therapy (CRT)) have a range of potential complications. The most common serious complications generally require surgical reintervention, where the wound is reopened to reposition a displaced lead or explant an infected system.^1^ Device reintervention can significantly impact on morbidity, mortality, the risk of subsequent complications and healthcare utilisation.^2^

To prevent the complications that lead to device reintervention, it is essential to understand the factors that influence their occurrence. We have previously shown that operator volume and seniority are associated with reintervention risk following permanent pacemaker (PPM) implantation.^3^ There are also data to suggest that operator and hospital volume, as well as physician specialty, are associated with outcomes following complex cardiac device implantation.^4^,^7^ However, most of these data are from North American sources and to our knowledge, there are no published data concerning operator seniority and outcomes with complex devices, which have higher rates of reintervention than single-chamber/dual-chamber pacemakers.

The aim of this study was to evaluate whether operator and hospital characteristics, and specifically operator volume and seniority, were associated with reintervention rates following a first permanent transvenous complex device implant.

## Methods

### Data source

This work was part of a quality improvement project on behalf of the UK National Audit of Cardiac Rhythm Management (NACRM), one of the domains of the National Cardiac Audit Programme managed by the National Institute for Cardiovascular Outcomes Research (NICOR). The UK Health Research Authority has approved NICOR’s collection and use of patient-identifiable data for audit, quality improvement and medical research.

National Health Service (NHS) hospitals in England are contractually required to submit details of cardiac rhythm management (CRM) procedures to the NACRM. This incorporates procedural information, including the device type, procedure type, implanting hospital and details of the doctor(s) who performed the procedure.

The study followed the Strengthening the Reporting of Observational Studies in Epidemiology reporting guideline for cohort studies.^8^

### Patient population

The study population was all patients undergoing their first transvenous complex device (ICD or CRT) implant between 1 April 2014 and 31 March 2019. To include a 1-year follow-up for all implants, data were extracted from all CRM device procedures in England submitted to NICOR between 1 April 2014 and 31 March 2020. This study period was chosen as the COVID-19 pandemic did not impact cardiac device procedures in the UK until afterwards.^9^ The extract contained no patient-identifiable data, but each patient’s NHS number (a unique identifier generated for each patient) was pseudonymised using one-way encryption. This allowed reinterventions to be identified whether these were performed at the original implanting centre or elsewhere in England.

Although hospitals in Scotland, Wales and Northern Ireland have contributed to the NACRM audit, this was incomplete during the study period, so their records were excluded, as were those from hospitals that did not submit data to NICOR in each of the years examined. We excluded individual records in which the device type could not be established, or the patient’s NHS number or the supervising consultant’s General Medical Council (GMC) number was missing.

### Study endpoints

The primary endpoint was the first device reintervention within the first year. Reinterventions comprised any of the following: lead revision (with or without a new generator), generator change alone, upgrade, downgrade, explant, pocket revision or haematoma evacuation. 1 year was chosen because reinterventions within this period can be reasonably attributed to complications of the initial implant (or the initial choice of device type). This aligns with the NICOR annual report, which details each hospital’s reintervention rate.^9^ Using the pseudonymised NHS number, we explored the data extract to identify patients who had a device reintervention.

### Definitions

We evaluated the following volumes and characteristics:

### Operators

For each procedure, the NACRM registry records first (scrubbed) operator’, ‘second (scrubbed) operator’ and ‘supervising consultant’ to take account of the fact that a proportion of cases are undertaken by trainees, supervised by a consultant. Doctors are identified using their GMC number. This unique identifier is issued to each doctor practising in the UK at the time of first GMC registration. The GMC register is publicly available, as is the date that each doctor joined the Specialist Register, which confirms completion of specialist training, and whether the doctor initially trained in the UK or elsewhere.

Supervising consultant volume: We used the British Heart Rhythm Society (BHRS) definitions to calculate each supervising consultant’s annualised transvenous complex device implant volume during the study period. This included any new transvenous complex device implant (ICD and CRT), as well as ICD/CRT upgrades where the functionality of the device was increased by the addition of an ICD and/or a left ventricular lead. Furthermore, based on BHRS definitions, a case was counted towards the total volume whether the supervising consultant was a ‘first operator’, ‘second operator’ or ‘supervising consultant’. To account for supervising consultants not implanting during the entire study period, we annualised implant volume by calculating the time between their first and last procedures on the database. To avoid overestimating annualised implant volume for low-volume supervising consultants, supervising consultants with less than a month on the database were rounded up to 1 month.

Supervising consultant seniority: From each Consultant’s GMC number, we identified how long they had been on the specialist register at the time of the procedure.

Supervising consultant country of initial medical qualification: UK versus non-UK.

Supervising consultant practising electrophysiologist: Whether the supervising consultant also appeared as a supervising consultant on the NICOR catheter ablation registry during the study period.

First operator status: In cases where a first operator GMC number differed from that of the supervising consultant, the first operator status was determined to be ‘specialist’ or ‘non-specialist’ based on whether the first operator was on the specialist register on the day of the procedure. Most ‘non-specialists’ implanting devices in the UK are doctors in cardiology training. However, a small number are non-consultant career-grade doctors, neither in training nor on the specialist register.

### Hospitals

Total volume: For each hospital, we calculated the annualised transvenous complex device implant volume during the study period. We used the same BHRS definitions of a ‘complex device’ implant described above.

Surgical centre: Whether cardiothoracic surgery was performed in the hospital during the study period.

### Data analyses and statistics

Categorical variables are expressed as a number (percentage) and continuous variables are not normally distributed as median (lower quartile to upper quartile). Categorical variables were compared using Pearson’s χ^2^ test, trends across categories using the linear-by-linear association test and continuous variables using the Mann-Whitney U test.

We used multilevel logistic regression to evaluate associations between supervising consultant and hospital volume/characteristics and 1-year reintervention. We constructed a hierarchical structure, in which hospitals and supervising consultants were included as random intercepts in mixed‐effects models to account for the clustering of patient outcomes within hospitals and supervising consultants.

We divided the continuous variables of interest (supervising consultant complex device volume, supervising consultant seniority and hospital complex device volume) into quartiles, such that each quartile contained a similar number of procedures. We first explored the relationship between individual variables and the study endpoints in unadjusted models. We then generated multivariable models, including all supervising consultant/hospital variables, with additional adjustment for implant year, device type, patient age, patient sex and aetiology of underlying cardiac disease. These additional variables were chosen as they may be associated with device complications, and complete data were available in the NACRM database.^10 11^ We performed subgroup analysis with cases grouped by device type (single/dual-chamber ICD vs CRT-pacemakers/defibrillators (CRT-P/D) devices). Lastly, we performed an additional analysis with adjustment for the supervising consultant’s annualised new PPM implant volume.

We performed a sensitivity analysis using the endpoint of lead revision at 1 year. For this analysis, cases were censored if they experienced reintervention for a cause other than lead revision. We also performed sensitivity analyses evaluating two different definitions of supervising consultant annual volume. (A) Supervising consultant ICD implant volume (including new/upgrade ICD implants) in an analysis including only single/dual-chamber ICDs; (B) supervising consultant CRT implant volume (including new/upgrade CRT-P/D implants) in an analysis including only CRT-P/D implants.

We analysed the relationship between supervising consultant seniority, evaluated as a continuous variable, and 1-year reintervention using restricted cubic splines in fully adjusted multivariable models.

We evaluated the current BHRS minimum volume standards for operators implanting complex devices (30 new/upgrade complex devices/year and 20 new/upgrade CRT devices/year for those implanting CRT devices).^12^ We also evaluated the equivalent Canadian Heart Rhythm Society guidelines (35 implant procedures per year of the most complex device type for which they were trained).^13^ Cases were grouped based on whether the supervising consultant fulfilled the minimum standard criteria, and the relationship with 1-year reintervention was evaluated in fully adjusted models as above.

The missing data rate for the variables included was low (0.2% of cases had one or more missing data points), so all available data were used for the analyses.^14^

All tests were two-tailed and a p<0.05 was considered significant. Analyses were performed using SPSS V.27.0 software package and R software, V.4.2.2 (R Foundation for Statistical Computing).

## Results

### Cohort derivation

**Figure 1:**
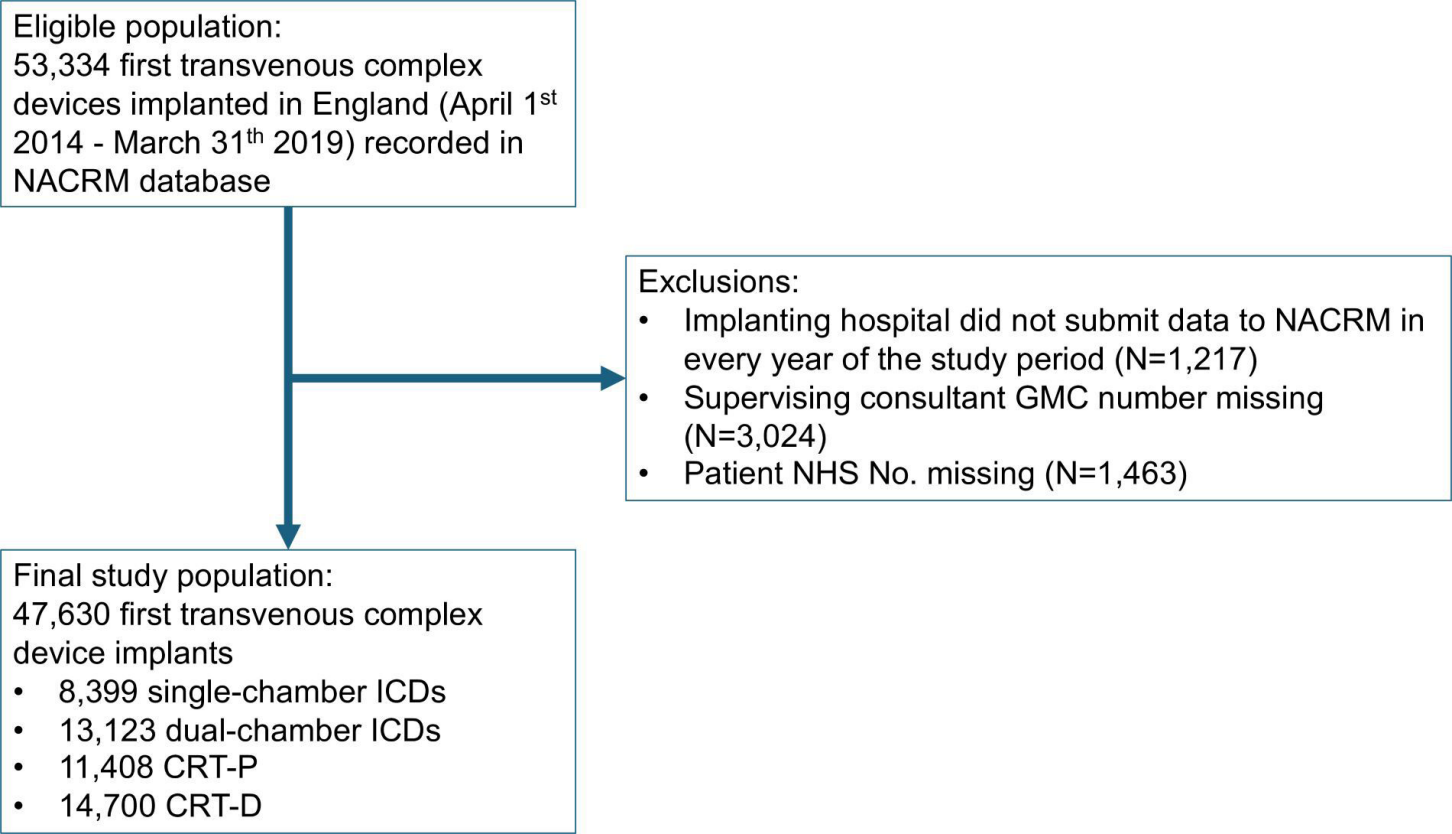
Derivation of the study population. CRT, cardiac resynchronisation therapy; GMC, General Medical Council; ICDs, implantable cardioverter defibrillator; NACRM, National Audit of Cardiac Rhythm Management; NHS, National Health Service.

During the study period, 53 334 first transvenous complex device implants were reported by NHS hospitals in England. We excluded 5704 cases (figure 1). This left 47 630 complex device implants in the analysis: 8399 (17.6%) single-chamber ICDs, 13 123 (27.6%) dual-chamber ICDs, 11 408 (24.0%) CRT-P devices and 14 700 (30.9%) CRT-D devices (table 1). The median patient age was 70.8 years and the majority were male (75.5%).

**Table 1:** Baseline characteristics of 47 630 first complex device implants included in the analysis

	Total (N=47 630)
Patient and device details	
Device type, N (%)	
Single-chamber ICD	8399 (17.6)
Dual-chamber ICD	13 123 (27.6)
CRT-P	11 408 (24.0)
CRT-D	14 700 (30.9)
Age (years), median (IQR)	70.8 (61.4–78.1)
No data, N (%)	3 (<0.1)
Sex, N (%)	
Male	35 965 (75.5)
Female	11 577 (24.3)
No data	88 (0.2)
Aetiology of cardiac disease, N (%)	
Coronary artery disease	20 092 (42.2)
DCM	10 426 (21.9)
Normal heart	2330 (4.9)
HCM	1532 (3.2)
Valve disease	939 (2.0)
Channelopathy	372 (0.8)
AVC	224 (0.5)
Sarcoid	180 (0.4)
Other	11 535 (24.2)
Operator characteristics	
Supervising consultant country of medical qualification UK, N (%)	39 780 (83.5)
Supervising consultant practising EP, N (%)	22 885 (48.0)
First operator on specialist register status, N (%)	39 836 (83.6)
Hospital characteristics	
Cardiothoracic surgical centre, N (%)	27 725 (58.2)

AVC, arrhythmogenic cardiomyopathy; CRT, cardiac resynchronisation therapy; CRT-D, CRT-defibrillators; CRT-P, CRT-pacemakers; DCM, dilated cardiomyopathy; EP, cardiac electrophysiologist; HCM, hypertrophic cardiomyopathy; ICD, implantable cardioverter defibrillator.

### Outcomes

The 1-year reintervention rate was 6.1% (N=2916) (table 2). The most common indication for reintervention was lead revision alone (4.5%, N=2122)

**Table 2:** Details of first reintervention at 1 year following 47 630 first complex device implants

Outcome	1-year reintervention
No reintervention, N (%)	44 714 (93.9)
First reintervention, N (%)	2916 (6.1)
Lead revision alone	2122 (4.5)
Lead revision+new generator	107 (0.2)
New generator alone	28 (0.1)
Device upgrade	290 (0.6)
Device downgrade	3 (0)
Wound revision/generator re-site	138 (0.3)
System explant	228 (0.5)

### Operator characteristics

There were 684 supervising consultants supervising device implants; their median total complex new implant volume (including all new and upgrade ICD/CRT implants) was 12/year (IQR 1–34) and median annualised total new implant volume (to also include new single/dual-chamber PPM implants) was 51/year (IQR 20–91).

200 of the 684 supervising consultants achieved the current BHRS minimum volume standard for complex devices (30 new/upgrade complex devices/year), and 176 the standard for CRT (20 new/upgrade CRT devices/year). Compared with those not meeting the BHRS complex device standard, supervising consultants meeting the standard were more likely to be qualified in the UK (85.0 vs 75.2%, p=0.005), more likely to practise cardiac electrophysiology (EP) (42.5% vs 33.7%, p=0.03) and be less senior (average date of entry on the GMC specialist register 3 years later, p<0.001).

7794 (16.4%) cases were performed with a first operator not on the specialist register (mostly trainees). The proportion of such cases increased with increasing supervising consultant seniority (14.2% of cases for Q1 vs 18.5% for Q4, p<0.001).

Procedures were performed in 119 different hospitals with a median total complex new implant volume of 72/year (IQR 12–148).

### Supervising consultant volume and outcomes

There was no difference in 1-year reintervention risk with increasing supervising consultant complex device volume (table 3). In an unadjusted analysis, the risk of reintervention in the highest volume quartile was no different from the lowest volume quartile (OR 0.91 Q4 compared with Q1; 95% CI 0.77 to 1.07, p=0.24). There were similar results in an adjusted model and when single/dual-chamber ICDs and CRT devices were analysed separately. Furthermore, with additional adjustment for supervising consultant annualised new PPM implant volume, the results were similar (adjusted analysis including all patients, OR 1.01 Q4 compared with Q1; 95% CI 0.83 to 1.22 p=0.96).

**Table 3:** The association between operator and hospital characteristics, and 1-year reintervention for first complex device implants

	Unadjusted		Adjusted*					
	All cases (N=47 630)		All cases† (N=47 539)	Single/dual-chamber ICD‡ (N=21 474)‡		CRT-P/D§ (N=26 065)		
	OR (95% CI)	P value	OR (95% CI)	P value	OR (95% CI)	P value	OR (95% CI)	P value
Operator characteristics								
Quartiles supervising consultant annualised complex device volume								
Q1 (<31 devices/year) (N=11 790)	1 (Reference)	–	1 (Reference)	–	1 (Reference)	–	1 (Reference)	–
Q2 (31–48 devices/year) (N=11 836)	1.06 (0.92 to 1.21)	0.43	1.05 (0.91 to1.21)	0.49	1.21 (1.03 to 1.42)	0.02	0.93 (0.77 to 1.13)	0.48
Q3 (49–67 devices/year) (N=12 069)	0.99 (0.86 to 1.15)	0.94	1.01 (0.87 to 1.17)	0.91	1.04 (0.87 to 1.23)	0.68	0.97 (0.80 to 1.18)	0.75
Q4 (>67 devices/year) (N=11 935)	0.91 (0.77 to 1.07)	0.24	0.89 (0.76 to 1.05)	0.17	1.00 (0.83 to 1.20)	0.97	0.84 (0.67 to 1.03)	0.10
Quartiles supervising consultant seniority								
Q1 (<4 years) (N=11 916)	1 (Reference)	–	1 (Reference)	–	1 (Reference)	–	1 (Reference)	–
Q2 (4–7 years) (N=11 903)	0.86 (0.76 to 0.96)	0.009	0.87 (0.77 to 0.98)	0.02	0.98 (0.83 to 1.16)	0.79	0.82 (0.70 to 0.96)	0.02
Q3 (8–12 years) (N=11 904)	0.80 (0.70 to 0.92)	0.001	0.81 (0.71 to 0.93)	0.003	0.87 (0.73 to 1.04)	0.13	0.79 (0.66 to 0.94)	0.008
Q4 (>12 years) (N=11 907)	0.92 (0.80 to 1.05)	0.19	0.93 (0.81 to 1.07)	0.31	0.99 (0.84 to 1.17)	0.93	0.93 (0.77 to 1.11)	0.42
Supervising consultant UK qualified (vs non-UK)	1.02 (0.88 to 1.28)	0.08	1.00 (0.86 to 1.16)	0.97	1.07 (0.89 to 1.29)	0.46	0.94 (0.78 to 1.14)	0.55
Supervising consultant practising EP	1.11 (0.99 to 1.24)	0.06	1.09 (0.97 to 1.23)	0.14	1.15 (0.99 to 1.32)	0.06	1.04 (0.89 to 1.21)	0.65
First operator non-specialist	0.96 (0.86 to 1.07)	0.48	0.99 (0.89 to 1.10)	0.84	1.00 (0.86 to 1.18)	0.96	0.98 (0.84 to 1.14)	0.81
Institution characteristics								
Quartiles annual hospital volume								
Q1 (<116 devices/year) (N=12 090)	1 (Reference)	–	1 (Reference)	–	1 (Reference)	–	1 (Reference)	–
Q2 (117–208 devices/year) (N=12 053)	1.09 (0.89 to 1.32)	0.41	1.07 (0.86 to 1.34)	0.48	1.32 (1.05 to 1.66)	0.02	0.98 (0.75 to 1.28)	0.88
Q3 (209–334 devices/year) (N=12 143)	1.10 (0.89 to 1.36)	0.36	1.06 (0.74 to 1.53)	0.71	1.14 (0.80 to 1.62)	0.48	1.06 (0.68 to 1.65)	0.80
Q4 (>334 devices/year) (N=11 344)	1.16 (0.91 to 1.49)	0.24	1.12 (0.76 to 1.65)	0.55	1.14 (0.79 to 1.66)	0.49	1.11 (0.69 to 1.77)	0.67
Cardiothoracic surgical centre	1.10 (0.94 to 1.28)	0.25	1.01 (0.75 to 1.37)	0.94	1.03 (0.76 to 1.39)	0.86	1.00 (0.69 to 1.46)	0.98

Data are presented for all devices and for procedures grouped by device type (single/dual-chamber ICD and CRT devices).

* Additional adjustment for implant year, device type, patient age, patient sex and aetiology of cardiac disease.

† 91 cases excluded due to missing data in one or more variables.

‡ 48 cases excluded due to missing data in one or more variables.

§ 43 cases excluded due to missing data in one or more variables.

EP, cardiac electrophysiologist.CRT, cardiac resynchronisation therapy; CRT-D, CRT-defibrillators; CRT-P, pacemakers; ICD, implantable cardioverter defibrillator.

We performed sensitivity analyses evaluating two alternative definitions of supervising consultant volume (table 4).

**Table 4:** Sensitivity analysis evaluating two alternative definitions of supervising consultant annual volume

Supervising consultant volume	Unadjusted		Adjusted* †	
	OR (95% CI)	P value	OR (95% CI)	P value
(A) ICD annual implant volume (including new/upgrade ICD implants)				
Analysis includes only single/dual-chamber ICD implants (N=21 522)				
Q1 (<12 devices/year) (N=5390)	1 (Reference)	–	1 (Reference)	–
Q2 (13–18 devices/year) (N=5409)	1.04 (0.88 to 1.23)	0.64	1.08 (0.91 to 1.28)	0.40
Q3 (19–26 devices/year) (N=5354)	0.95 (0.79 to 1.13)	0.54	0.98 (0.82 to 1.17)	0.82
Q4 (>27 devices/year) (N=5369)	0.98 (0.81 to 1.18)	0.81	1.04 (0.86 to 1.25)	0.72
(B) CRT annual implant volume (including new/upgrade CRT implants)				
Analysis includes only CRT-P/D implants (N=26 108)				
Q1 (<21 devices/year) (N=6454)	1 (Reference)	–	1 (Reference)	–
Q2 (22–31 devices/year) (N=6563)	0.96 (0.81 to 1.14)	0.66	0.97 (0.81 to 1.17)	0.78
Q3 (32–46 devices/year) (N=6541)	0.93 (0.77 to 1.11)	0.40	0.93 (0.77 to 1.13)	0.46
Q4 (>46 devices/year) (N=6550)	0.77 (0.62 to 0.95)	0.01	0.79 (0.63 to 0.98)	0.03

(A) Supervising consultant ICD implant volume (including new/upgrade ICD implants) in an analysis including only single/dual-chamber ICDs; (B) supervising consultant CRT implant volume (including new/upgrade CRT-P/D implants) in an analysis including only CRT-P/D implants. For each volume definition, data are presented for unadjusted and adjusted analyses.

* Adjusted for implant year, device type, patient age, patient sex, aetiology of cardiac disease, supervising consultant seniority (quartiles), supervising consultant UK qualified, supervising consultant practicing EP, first operator non-specialist, hospital annual implant volume (quartiles) and cardiothoracic surgical centre.

† 91 cases excluded due to missing data in one or more variables for ICD analysis and 43 cases for CRT analysis.

CRT, cardiac resynchronisation therapy; CRT-D, CRT-defibrillators; CRT-P, CRT-pacemakers; EP, cardiac electrophysiologist; ICD, implantable cardioverter defibrillator.

In an analysis including only single/dual-chamber ICD implants (N=21 522) and defining supervising consultant annual volume as new/upgrade ICD implants per year, there was no difference in 1-year reintervention risk with increasing supervising volume in unadjusted or adjusted analyses (table 4).

We performed an analysis including only CRT-P/D implants (N=26 108) and defining supervising consultant annual volume as new/upgrade CRT implants per year. There was an association between operator volume and 1-year reintervention, but this was of borderline statistical significance and only evident in the highest compared with the lowest volume quartile of operators (adjusted OR 0.79, 95% CI 0.63 to 0.98, p=0.03) (table 4).

### Supervising consultant seniority and outcomes

There was a non-linear relationship between 1-year reintervention risk and supervising consultant seniority (table 3). In an unadjusted analysis, there was no difference in risk of reintervention between the quartiles of most and least senior operators (OR 0.92 Q4 compared with Q1; 95% CI 0.80 to 1.05, p=0.19). However, both the second (OR 0.86 Q2 compared with Q1; 95% CI 0.76 to 0.96, p=0.009) and third (OR 0.80 Q3 compared with Q1; 95% CI 0.70 to 0.92, p=0.001) quartiles had a lower reintervention risk than the first quartile. Similar findings were observed in an adjusted model. When we evaluated device types separately, the relationship remained significant only for CRT devices but not single/dual-chamber ICDs (table 3).

**Figure 2:**
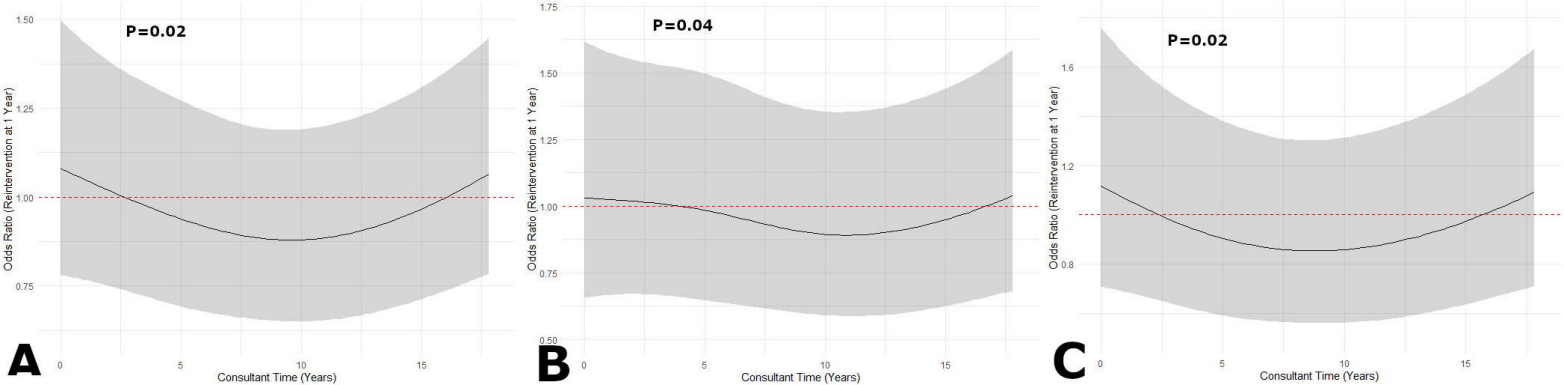
The association between supervising consultant seniority, quantified as time on the specialist register on the day of the procedure, and 1-year reintervention risk. Data are presented separately for (**A**) all cases, (**B**) single-chamber and dual-chamber ICDs and (**C**) CRT-P and CRT-D devices, using a restricted cubic spline regression model. Data were fitted by a logistic regression model, and the model was conducted with four knots at the 5th, 35th, 65th and 95th percentiles of operator volume (reference is the 5th percentile). Solid lines indicate ORs, and shadow shape indicates 95% CI. Adjustment is made for implant year, device type, patient age, patient sex, aetiology of cardiac disease, supervising consultant annual implant volume (quartiles), supervising consultant UK qualified, supervising consultant practising EP, first operator non-specialist, hospital annual implant volume (quartiles) and cardiothoracic surgical centre. CRT, cardiac resynchronisation therapy; CRT-D, CRT-defibrillators; CRT-P, CRT-pacemakers; EP, electrophysiologist; ICDs, implantable cardioverter defibrillator

When supervising consultant seniority was evaluated as a continuous variable, in an adjusted analysis (adjusted for operator/hospital variables, implant year, device type, patient age, patient sex and aetiology of underlying cardiac disease), there was a similar non-linear relationship between seniority and 1-year reintervention (figure 2). The risk of reintervention reduced with increasing operator seniority, until an operator had been on the specialist register for 8–10 years, after which risk started to increase again. This finding was most marked with CRT devices.

### Additional supervising consultant characteristics and outcomes

We evaluated three additional operator characteristics (table 3): whether the supervising consultant’s initial medical qualification was in the UK, whether the supervising consultant was a practising EP and whether the first operator was on the specialist register. None of these variables were associated with 1-year reintervention.

### Institutional volume and characteristics and outcomes

There was no difference in the risk of 1-year reintervention with increasing annualised centre new complex device volume or the hospital having on-site cardiothoracic surgery (table 3).

### Minimum volume standards for supervising consultants and outcomes

36 098 of 47 630 complex devices (75.8%) were implanted by a supervising consultant achieving the current BHRS recommended minimum volume standard for complex devices (30 procedures/year).^12^ In an adjusted analysis, there was no difference in 1-year reintervention with these implants compared with those implanted by a supervising consultant not achieving the standard (OR 1.00; 95% CI 0.89 to 1.23, p=0.99). The results were similar when procedures were grouped by operator seniority: including the most and least senior operators (Q1 and Q4, N=23 823) (OR 0.95; 95% CI 0.81 to 1.12; p=0.56) and operators in the middle two quartiles of seniority (Q2 and Q3, N=23 807) (OR 1.02; 95% CI 0.86 to 1.21; p=0.83).

20 078 of 26 108 CRT (76.9%) devices were implanted by a supervising consultant achieving the current BHRS minimum volume standard for CRT. In an adjusted analysis, there was no difference in 1-year reintervention for these implants compared with those implanted by a supervising consultant not achieving the standard (OR 0.94; 95% CI 0.80 to 1.08, p=0.40).

Similar results were obtained using the Canadian Heart Rhythm Society guideline recommendation for maintaining competence (35 complex implants/year).^13^ 166 of 684 supervising consultant operators achieved this volume, implanting 32 440 of 47 630 complex devices. In an adjusted analysis, there was no difference in 1-year reintervention for these implants compared with those implanted by a supervising consultant not achieving the standard (OR 0.96; 95% CI, 0.86 to 1.08=7, p=0.47).

### Sensitivity analysis

We performed a sensitivity analysis using the alternative endpoint of lead revision at 1 year. The results for all analyses were similar to those using the endpoint of any 1-year reintervention (data not shown).

## Discussion

We evaluated the relationship between operator and centre characteristics and the risk of reintervention, in a nationwide study of 47 630 first transvenous complex device implants. There are two findings of note. First, there was a U-shaped relationship between supervising consultant seniority and reintervention risk, with the most and least senior operators having a similar reintervention risk, but procedures performed by operators in the middle two quartiles of seniority having a lower reintervention risk. This finding was most pronounced with CRT devices. Second, neither supervising consultant nor centre operative volume was associated with reintervention risk.

To our knowledge, there have been no previous studies assessing the relationship between operator seniority and outcomes with complex devices. We have previously shown that PPM implants where the first operator is a trainee have worse outcomes than implants performed by an operator who has finished their training.^3^ The finding of a U-shaped relationship between operator seniority and 1-year reintervention suggests that with increasing experience physicians may continue to improve their operative skill for some years after finishing their training. Conversely, their outcomes appear to worsen towards the end of their career with a similar operative risk to newly qualified consultants.

A number of potential explanations exist for this finding. More senior operators may simply have diminished sustained attention, visuospatial abilities and manual dexterity, factors that may in part underlie our findings.^15^,^17^ There is an existing literature in other procedural specialities, evaluating the operator seniority-outcome relationship. Many studies have demonstrated operator learning curves, with worse procedural outcomes in the most junior operators, that improve over time in practice.^18 19^ In contrast, some studies have also described a decline in outcomes in the most senior operators.^20 21^

Casemix and/or involvement of trainees may provide alternative explanations for the finding of increased reintervention rates for the most senior consultants. Although we adjusted for basic patient demographics, it is possible that the most senior and/or most junior supervising consultants performed the most difficult cases, leading to higher reintervention rates. We found that the proportion of cases performed by a first operator not on the specialist register increased with greater operator seniority. Although we adjusted for the seniority status of the first operator, it is possible that some of our findings are explained by the greater involvement of trainees in procedures performed by the most senior supervising consultants, leading to worse outcomes.

Our findings concerning the seniority-outcome relationship for complex CIED have several implications. First, newly qualified consultants may benefit from mentoring, especially around CRT devices. Second, all operators, irrespective of seniority, should continuously evaluate their outcomes and share them within their centre and more widely through national audit.

Although in a sensitivity analysis there was an association between supervising consultant annual CRT volume and reintervention, in our main analyses neither supervising consultant nor hospital volume were associated with reintervention. This is at variance with previous studies.^4^,^6^ Several factors may have contributed to this finding. First and most importantly, procedure volumes in our analysis were significantly higher than those in previous studies.^4^,^6^ In our analysis, the lowest volume quartile for operators was <31 devices/year and centres <116 devices/year. In comparison, in the analyses by Freeman *et al*, the respective volumes were <4 devices/year and <24 devices/year.^4 5^ Second, previous studies used older data.^4^,^6^ It is likely that implant techniques, device technology and clinical practice have evolved since these early studies, which may have reduced complication rates. Third, previous studies used predominantly North American data, where devices may be implanted by cardiothoracic surgeons, who may have higher complication rates.^7^ In the UK, transvenous complex devices are implanted exclusively by cardiologists. Lastly, previous studies focused on early complications, whereas we assessed 1-year reintervention.^4 6^

Our data do not indicate that operative volume is not an important predictor of procedural risk. Our findings suggest that, at the operative volumes observed in our analysis, which reflect those of implanters in England, there is little difference in reintervention risk between higher-volume and lower-volume operators. Using the same NICOR dataset, we have previously demonstrated a lower reintervention rate following PPM implantation with higher-volume operators.^3^ However, the difference in reintervention risk was primarily observed in the lowest volume quartile compared with higher volumes, and there was little difference in risk between the three higher volume quartiles. In the current analysis, very few of the procedures were performed by operators that would have been in the lowest quartile of PPM implants in our previous study. We feel it is therefore likely that the discrepancy in results, and the lack of association between outcomes and volume in the present study, is primarily due to the two studies focusing on different parts of the volume-outcome curve, with the current analysis evaluating the higher volume end of the curve where the difference in risk between volumes is small.

The current BHRS minimum volume standards for operators implanting complex devices (in place throughout the study period) are 30 new/upgrade complex devices/year and 20 new/upgrade CRT devices/year for those implanting CRT devices.^12^ The majority of procedures were undertaken by consultants meeting these standards, and this may not have been the case had they not been in place. While we found no clear association between outcomes and overall complex procedure volumes for operators or centres, there was a clear trend for improved CRT outcomes as CRT volume increased. This may reflect the significantly greater technical challenges in CRT implantation compared with ICD and pacemaker procedures. It may be that the advent of conduction system pacing will result in a fall in CRT procedures, and that CRT implantation should be restricted to a smaller number of operators in order to maintain standards.

We found that being an ablating EP did not influence reintervention rates. This contrasts with a previous study by Chui *et al* which found EP had a lower reintervention risk than other implanters. Several factors may explain this disparity.^7^ First, Chui *et al* used medical certification to define a doctors’ subspecialty rather than whether they perform catheter ablations. Second, in the UK, many doctors train with device implantation as their subspecialty. Many of the non-EP in our analysis will likely have a specific subspecialty interest in device implantation (eg, they have trained as heart failure/device specialists rather than ablating EP). However, in the UK, subspecialty training is not recorded in the GMC specialist register, and therefore, it is not possible to assess this in our study.

This study is one of the largest to report outcomes following complex device implant in a socialised healthcare system and has several strengths. First, it includes data from most complex device implants performed in the NHS in England over 5 years. Second, it includes patients of all ages and, in contrast to some previous studies, is not limited to a specific healthcare provider. Third, we used a unique identifier (the pseudonymised NHS number) to identify reintervention in hospitals other than the original implanting centre.

### Limitations

This study has several limitations. First, detailed data concerning patient comorbidity and procedural urgency are not collected in the NACRM database. These factors may influence reintervention rates and confound our findings.

Second, mortality data are not collected as part of the NACRM database. Many complex devices are implanted in patients with heart failure who are at increased risk of death, and this may have had an impact on our primary endpoint of 1-year reintervention. Furthermore, data on other important complications, such as pneumothorax and bleeding, are not collected in the database.

Third, due to missing data, we excluded 5704 (10.7%) cases from our analysis. Among the patients we included, 91 (0.2%) had data missing in one or more of the analysed variables.

Fourth, given that our extract contained no patient-identifiable data, it was not possible to validate our data by review of patients’ notes in individual hospitals.

Fifth, we have not analysed data from private hospitals due to low data completeness. Some doctors may implant devices in private hospitals, which could lead to our analysis underestimating their true operative volume. However, most implanting centres in England are NHS hospitals, and this is therefore unlikely to have a significant impact on our results.

Sixth, we categorised first operators as specialists or non-specialists based on whether they were on the GMC specialist register at the time of the implant. Although most non-specialist first operators are trainees, a small number will be non-consultant career-grade doctors, who are neither in training nor on the specialist register. From our data, it is impossible to differentiate between these groups. Furthermore, some supervising consultants enter the GMC specialist register after a period of practice as a consultant in another country. For these operators, time on the GMC specialist register will underestimate their true seniority. However, we feel this is likely to affect only a small number of operators.

Seventh, we could not identify reintervention procedures performed in hospitals outside of England. While this is likely to account for only a small number of patients, it is a limitation.

Eighth, we have no data concerning the reason for device upgrade or downgrade, and whether they were performed due to device malfunction or a change in the patient’s clinical status.

Lastly, as this is an observational study, it is not possible to determine a causal relationship between outcomes and supervising consultant characteristics.

## Conclusions

There is a U-shaped curve between operator seniority and reintervention risk for complex device implantation, with operators in years 4–12 at the lowest risk. In contrast, procedure volume or hospital characteristics were not associated with reintervention risk. Although there are several potential explanations, these data suggest that while newly qualified consultants may benefit from mentoring, all operators should continuously evaluate their outcomes and share them within their centre and more widely through national audit.
